# Women’s use of non-conventional herbal uterotonic in pregnancy and labour: evidence from birth attendants

**DOI:** 10.1186/s12884-022-04934-2

**Published:** 2022-07-27

**Authors:** Joshua Sumankuuro, Leonard Baatiema, Judith Crockett, Jeanine Young

**Affiliations:** 1 Faculty of Public Policy and Governance, Simon Diedong Dombo University of Business and Integrated Development Studies, Wa, Ghana; 2 Centre for Health Policy, School of Public Health, Faculty of Health Sciences, University of the Witwatersrand, Johannesburg, South Africa; 3 School of Community Health, Faculty of Science, Charles Sturt University, Orange, New South Wales Australia; 4 School of Public Health, Faculty of Community and Health Sciences, University of the Western Cape, Cape Town, South Africa; 5 Department of Health Policy and Management, University of Ghana, Accra, Ghana; 6 Department of Global Health and Population, Harvard T.H Chan School of Public Health, Harvard University, Boston, USA; 7 School of Nursing, Midwifery and Paramedicine, University of the Sunshine Coast, Sippy Downs, Queensland, Australia

**Keywords:** Maternal and neonatal health, Pregnancy and childbirth, Traditional birth attendants, Herbal uterotonics, Low-and middle-income settings

## Abstract

**Background:**

Over the years, governments and stakeholders have implemented various policies/programmes to improve maternal health outcomes in low-middle-income countries. In Ghana, Community Health Officers were trained as midwives to increase access to skilled maternal healthcare. The government subsequently banned traditional birth attendants from providing direct maternal healthcare in 2000. Despite these, there is an unprecedented utilisation of TBAs’ services, including herbal uterotonics. This has attempted to defeat stakeholders’ campaigns to improve maternal health outcomes. Thus, we explored and highlighted *herbal uterotonic consumption in pregnancy and birth and the implications on maternal and newborn health outcomes in North-Western Ghana.*

**Methods:**

This was an exploratory qualitative study that investigated traditional birth attendants (*n* = 17) and healthcare providers' (*n* = 26) perspectives on the intake of herbal uterotonics in pregnancy and childbirth in rural Ghana, using in-depth interviews. A combination of convenience, purposive and snowball sampling procedures were employed in selecting participants.

**Results:**

Findings were captured in two domains: (1) perceived rationale for herbal uterotonic intake, and (2) potential adverse impacts of herbal uterotonic intake in pregnancy and labour, and nine topics: (i) confidence in unskilled attendance at birth, (ii) cost and a shortage of essential medicines, (iii) herbal uterotonics as a remedy for obstetric problems, (iv) herbal uterotonics facilitate birth, (v) attraction of home birth for cultural reasons, (vi) affordability of herbal uterotonics, (vii) unintended consequences and adverse outcomes, (viii) risks using herbal uterotonics to manage fertility and (ix) risks using herbal uterotonics to facilitate home birth*.*

**Conclusion:**

The findings have suggested that the intake of non-conventional herbal uterotonic is widespread in the study area, although the constituents of the herb are unknown. However, complex and multiple factors of healthcare cost, desire for homebirth, unawareness of the negative effects of such substances, perceived way of addressing obstetric problems and cultural undertones, among others, accounted for herbal uterotonics consumption. We also encourage research into the constituents of ‘mansugo’ and the potential benefits and adverse effects. We recommend qualitative studies involving previous users of this herbal uterotonic to inform policy and healthcare provision.

## Introduction

For decades, maternal mortality has been a public health challenge in low- and middle-income countries (LMICs) [1, 2]. Research has shown that sub-Saharan Africa and South Asia accounted for 66% (196000) and 86% (254000), respectively, of the estimated global maternal deaths in 2017 alone [1]. Similarly, in a review of verbal autopsy data on Ghana, Sumankuuro and colleagues reported the maternal mortality ratio to be 380 per 100,000 live births [1, 2]. In terms of birth rate, Ghana recorded declining birth rates (per 1000 inhabitants) of 30.28, 29.84, 29.41 and 28.99, respectively for 2016, 2017, 2018 and 2019 [3].

Factors such as increased use of unauthorised herbal medicines, unskilled birth care and deplorable health system issues were associated with maternal health outcomes in Ghana. The Government of Ghana has thus implemented several initiatives to address this menace of avoidable maternal morbidity and mortality, most notably, free healthcare during antenatal, perinatal, and the early postnatal periods [4–6]. Further to this, the fee-exemption policy of Ghana’s National Health Insurance Scheme introduced in July 2008; the skills upgrade of Community Health Nurses and posting of same to Community-based Health Planning and Services compounds were specific initiatives to improve maternal healthcare access, reduce healthcare costs and increase access to skilled care [6, 7]. Despite these policies, the challenges in maternal health service delivery in Ghana remain high. Crucial among the issues relates to the concomitant use of non-conventional herbal uterotonics [7, 8].

A particular herbal uterotonic that is predominantly used in northern Ghana and the associated health implications from the usage are the focus of this study. The herbal uterotonic (known as “*mansugo*” among sections of northern Ghana) is a collective term used by the participants in this study to refer to “herbal concoction or an herbal mixture or herbal product” provided by traditional birth attendant (TBA) to women during pre-pregnancy, pregnancy and labour. The World Health Organisation (WHO) has defined a TBA as *a person who assists a mother during childbirth and who initially acquired her skills by delivering babies herself or through apprenticeship to other traditional birth attendants* [9]*.*

Broadly, *herbal uterotonics* include herbs, herbal materials, herbal preparations, extracts from plants and are consumed unprocessed/unrefined by pharmaceuticals and applied during pregnancy and labour, to promote safe pregnancy and expedite childbirth or self-abort a pregnancy [10]. Herbal uterotonic is classified as herbal medicine. The World Health Organisation (WHO) defines traditional or alternative medicines as a broad set of healthcare practices that are not part of that country’s tradition and are not integrated into the dominant health care system [11]. Historically, herbal uterotonic use was beneficial to pre-pregnant women and pregnant women [12]. In many countries, they have been used to treat multiple pregnancy-related problems, including facilitating pregnancy, pregnancy prevention, induction of labour, the expulsion of retained matter, and self-manage abortion [13]. Notably, herbal uterotonics are most used by less educated women in low-income countries [14, 15].

Despite widespread use, the safety of the herbs consumed by pregnant women in low-middle-income regions such as sub-Saharan Africa is not well-established [16, 17] . Furthermore, the evidence has shown that the combined use of herbal uterotonics and conventional/allopathic medicines may expose pregnant women to substantial risks of obstetric complications [12]. For example, studies in Ghana and Uganda have demonstrated that postpartum haemorrhage was associated with herbal uterotonics consumption in pregnancy [18]. Similarly, Sumankuuro and colleagues reported neonatal deaths among pregnant women who used herbal uterotonics during labour [19].

Despite these issues, ongoing staff shortages and inadequate resources at primary healthcare (PHC) facilities, increased waiting time during antenatal visits, poor treatment by nurses during health facility birth, and increased hospital referrals remain problematic [8, 19]. These barriers influence women and families’ ongoing decisions to utilise the services of TBAs [8]. Historically, TBAs were specifically roped into Ghana’s health system after receiving some training in; basic live-saving, infection control, and effective management of labour [9]. Over forty years ago, it was recognised in many countries that it was possible to utilise and educate TBAs due to the shortage of trained healthcare providers. Based on these issues, we aimed to explore women’s herbal uterotonic use and the implications on maternal and newborn health outcomes in the North-Western Ghana.

### Research context

Although the area lies within the Guinea and Sudan Savanna Zones with sparse and very few tree species, herbalists, traditional healers, and traditional birth attendants (TBAs) obtain their plant products from within the region [20]. In addition, a few practitioners with operational areas in Central to Southern Zones of Ghana may acquire herbs/plant extracts that are not found in the Upper West Region [15, 21]. Within the study area, TBAs mostly obtain their plant extracts from the mistletoe plant (*Viscum album* of the family Santalaceae), parasitic plants (mainly on the stem) on the dawadawa tree (*Parkia biglobosa*, also known as the African locust bean from the Fabaceae family), among other plants with potential medicinal properties [20, 22].

Herbal uterotonic (‘local herbal uterotonic’) is mainly mixed with a drink for oral intake; some types are mixed with body lotion/pomade for smearing on the pregnant woman’s abdomen or inserted vaginally, to induce labour [10].

Two main kinds of herbal uterotonic were identified in this study: those for prenatal care (“cold *mansugo*”) and others used to induce labour (called “hot *mansugo*”). ‘Cold herbal uterotonic’ was administered orally during pregnancy as prenatal care medicine, while the ‘hot herbal uterotonic’ was administered per vagina, during labour to facilitate birth [19]. It must be noted that some women administer the herbal uterotonic before seeking care at the health facility, have home birth alone or in the presence of relative/s and some receive the herbal uterotonic when they seek birth care from the TBAs.

## Material and methods

### Research setting

The study was conducted at Nadowli-Kaleo and Daffiama-Bussie-Issa districts of the Upper West Region (UWR) of Ghana. Of the population aged 11 years and over, in Nadowli-Kaleo, only 51% of the population were classified as literate (53% males vs 47% females). In contrast, in Daffiama-Bussie-Issa District, only 42.3% met the literacy criteria (48.2% males vs 37% females. Broadly, residents had very low secondary or tertiary level education (males 5%, females 2.8%) [23, 24].

Nadowli-Kaleo and Daffiama-Bussie-Issa districts are impoverished areas dominated by subsistence farming. More than 80% of the population (estimated population of 98,000 people) had no formal sector employment [23, 24]. Most of the population comprises youth (15–35 years) who live on less than the Ghana Cedi equivalent of five United States dollars a day.

### Research design

This exploratory qualitative study investigated herbal uterotonic uptake by women in the Nadowli-Kaleo and Daffiama-Bussie-Issa districts in the Upper West Region (UWR) of Ghana from the perspectives of TBAs and healthcare providers.

### Study population

Healthcare providers (*n* = 26) and traditional birth attendants (*n* = 17) participated in the study. The healthcare providers comprised of, a Physician, a Pharmacist, Midwives, Community Health Nurses, and Enrolled Nurses. All participants were between the ages of 18 to 70 years. Overall, 43 participants (*n* = 40 female, *n* = 3 male) were included in the study.

### Sampling and recruitment procedures

A combination of convenience, purposive, and snowball sampling approaches was used in selecting participants for the study. Each participant participated in an individual in-depth interview (IDI). Under the snowballing approach, we first identified a TBA through the Community-based Health Surveillance Volunteers (CHSVs). This TBA referred the research team to other TBAs, that resided within the study communities. Overall, twenty TBAs were identified, and seventeen gave consent and participated in the study. Cultural reasons were cited for declining the consent to participate in the study. Information sheets containing the study aims, and data use, were given to the healthcare providers in selected health facilities. After reading, those who gave consent to participate were contacted and interviews were arranged at a mutually agreeable time. The Health and Medical Directors were invited to participate through a specific letter of invitation.

### Research instruments

A semi-structured interview topic guide was used in collecting data. The guide was prepared based on the evidence in the literature and informed by cultural knowledge and previous experiences of members of the research team. In addition, the interview topic guide content was checked by members of the research team, including experienced researchers and academics. As a result, the content of the instruments was the same for both participant groups. Table 1 contains the interview topic guide.

**Table 1:** Interview guide

Interview topics	
• Perceived benefits of using uterotonic substances • Perceived risk of using the uterotonic substance • Indications for use and dosage • Knowledge and experience with local herbal uterotonic/uterotonic substance • Traditional birth attendant motivation for maternal care and uterotonic use.	

### Data collection

Individual face-to-face interview techniques were used to collect data from all participants. The questioning focused on the topic areas presented in Table 1. Using the two types of participants helped to explore and cross-validate perspectives that motivated the utilisation of the herbal uterotonics and uptake of TBA services during pregnancy, labour, and birth.

We interveiwed TBAs in the local language (*Dagaare*) and English for the Healthcare Providers, between October 2020 and June 2021. Interviews were audiotaped and transcribed verbatim. Interviews lasted between 15 to 20 minutes.

### Data processing and management

Three field assistants with a minimum of an undergraduate degree in social science and public health fields who were proficient in the local language “Dagaare,” were recruited and trained by the researchers for a week on ethics in research, questionnaire administration, data integrity, and confidentiality issues of participants. The training included interpretation of interview questions and data management.

To achieve the data’s accuracy and dependability, all audio recordings were first transcribed (hand-written) in “Dagaare” and then translated into English. Two language experts at the Ghana Institute of Languages were engaged to validate the transcriptions and translations. Transcripts were exported into qualitative data management software (NVivo version 7.5) for coding. A coding framework was developed to code the text. Both computerised and manual coding was used. The computerised coding was complemented by topics identified in the manual coding process and professional experience. During the manual coding, data were thoroughly read and re-read to identify domains and themes. The research team conducted the coding independently and reconciled any differences that emerged. Patterns in the codes were identified and grouped into topics [25]. The topics were subsequently summarised into domains based on similarities, the content, and the meaning. Participants quotes were used to support the topics.

### Data analysis

Qualitative data arising from open-ended interview questions were transferred into NVivo™ software and analysed using Gibbs’s framework, which entails transcription and familiarisation, code building, theme development, and data consolidation and interpretation [26]. The data analysis involved prolonged engagement with the data. After each interview, notes were made. Emergent issues on herbal uterotonics (local herbal uterotonic) were grouped as factors and broad themes from the interview transcripts, written notes, and researchers’ reflections. The research team discussed emergent codes and organisation of themes to reach a consensus of themes and to manage dissenting findings. The views of TBAs and health providers were then grouped.

### Quality control

The trustworthiness of the study was achieved using several procedures: investigated (member checking) until saturation was achieved; prolonged engagement with each participant; field notes were also taken to record non-verbal cues/observations during the interviews and then independent coding and checking of transcripts ensured that the data and analysis were credible.

## Results

Two key domains, 1) perceived rationale for herbal uterotonic intake and 2) potential adverse impacts of herbal uterotonic intake in pregnancy and labour, were identified from the analysis, each with relevant themes. Under each domain, findings from the analysis are presented using these identified topics.

### Perceived rationale for herbal uterotonic intake

Six primary reasons for herbal uterotonic intake were identified: (i) *confidence in unskilled attendance at birth*, (ii) c*ost and a shortage of essential medicines*, (*iii) herbal uterotonics as remedy for obstetric problems*, (iv) *herbal uterotonics facilitate birth*, (*v) attraction of home birth for cultural reasons, (vi) affordability of herbal uterotonics.*

i) Confidence in unskilled attendance at birth

TBAs have long provided herbal uterotonics to expectant mothers and continue to offer. TBAs believed that having ready access to herbal uterotonic was a crucial factor that shaped women’s preference for their maternal healthcare services. Part of the attraction lies in the belief that herbal uterotonics are more potent and effective in promoting safety in pregnancy and facilitating active labour than the allopathic or orthodox uterotonics.

*“If they even come here (health facility) they will still go and do their black magic to see whether the baby inside is actually a human being. Whether the outcome will be positive. Those are some of the things – that’s why they still patronise their services. The women believe the TBAs are spiritually strong, not that she only palpates but she’s also like a soothsayer. She also foretells the future occurrence. That is their confirmation point. They believe that she has ‘four eyes’, and she sees double. That’s why they go there. If they even go and do scanning and the scan says the baby is in breech position, they will go there, that she’s capable of repositioning the baby to cephalic. They will go there for the palpation so that she can turn the baby into cephalic and they will come and deliver per vagina instead of caesarean section” [Midwife, 42 years].*

Besides the observation of the Midwife, a TBA has praised the efficacy of their services as:

*“We have herbal uterotonics administered to expectant mothers to ensure safe pregnancy and smooth childbirth. The product is more potent for labour than what is given at the clinic now. The herbal uterotonic is a multipurpose herb. It has the potency to stop threats of miscarriage and other complications. Also, labour progresses very fast when the “hot” herbal uterotonic is taken orally. We, however, wished the government could endorse it for expectant mothers to administer it freely” [TBA, 62 years].*

ii) Cost and shortage of essential medicines

Midwives reported that they were often unable to provide appropriate quality care, particularly if medicines were required. During the interviews, participants raised the issue that the National Health Insurance Scheme (NHIS) excluded allopathic uterotonics in the national essential medicine list for community-based health planning and services (CHPS) health facilities. This presented a substantial challenge in conducting deliveries, especially when there is postpartum haemorrhage (PPH) or if there is a need to expel post-birth retained matter.

*“Yes, especially, uterotonic [allopathic], which is very important to give to the woman immediately or within the first few minutes of birth, but health insurance has made it impossible for CHPS facilities to provide uterotonic [allopathic] post-birth, even though these medicines also prevent PPH. It can even contract the uterus and help expel the retained products – the placenta and other matter” [Midwife, 29 years].*

In the case of the health centres in Ghana, other health professionals noted that medical suppliers were often reluctant to restock essential medicines in health facilities due to delayed reimbursements for claims for services by the National Health Insurance Authority (NHIA).

*“If you do not pay at least part of your debt, they [suppliers] will not give you the drugs [medicine]. Besides, sometimes, when we go to the Diocesan Pharmacy and do not find uterotonic drugs, it is all because of our indebtedness to them. Meanwhile, we need these essential medicines to treat maternal cases” [Midwife, 32 years].*

Another healthcare professional highlighted the significant problems in safely managing birth if medicines were required:

*“If we manage to give the drugs during labour, the health insurance will not pay. As health centres, we are required to refer them. With what we are having here, I think at our level it’s okay. We have magnesium sulphate, that one we can use… If the blood pressure (BP) is going higher, we can use Nifedipine, but the Dexamethasone we must refer to Nadowli and other infections associated with childbirth, we cannot give medicines. The truth is that we must refer to the hospital” [Midwife, 36 years].*

Overall, the prescribing policy for lower-level primary healthcare (PHC) facilities such as community-based health planning and service (CHPS) compounds and health centres, under the National Health Insurance Scheme (NHIS), partly accounted for both cost and access problems.

*“The NHIS has removed some essential medicines, such as antibiotics. They have been taken out of the approved drug list because CHPS compounds are small facilities. If we prescribe it, they (NHIA) will not pay. Therefore, we sell it to them (mothers/families), which they always complain they do not have money to pay for medicines” [Healthcare provider, 35years].*

Both posed challenges for pregnant women to obtain relevant medicines from those facilities when they seek care. Herbal uterotonic was an obvious choice for pregnant women because of the lower price compared to prescribed modern interventions and medication from a health facility.

*“Pregnant women’ resort to traditional medicines because of the cost of fuelling someone’s motorbike to send them [pregnant women] to hospital, health screening, and ANC classes. Also, anytime they sought care, drugs, basic essential items, and medicines such as ringers’ lactate, and other infusions were prescribed for them to buy. Even with the active NHIS subscription, only the older mothers usually benefited. Still, all medicines are prescribed for them to purchase from ‘over the counter licensed medicine sellers’ or pharmacy shops” [Healthcare provider, 48 years].*

iii) Herbal uterotonic as remedy for obstetric problems

TBAs identified many perceived benefits from herbal uterotonics for the expectant mother and her baby. For example, TBAs reported that the perceived benefits of “cold” herbal uterotonic included treating fibroids, correcting breech presentations, and other related obstetric issues.

*“When the baby is in breech presentation, and then I grind the “charcoal-like” herbal uterotonic and mix it with a drink for her to drink, it corrects [repositions the baby to cephalic] it. Some of the problems in the womb are usually the cause of complications. Therefore, it will improve her health condition when a pregnant woman administers it according to my [TBA’s] prescribed dosage. Also, I often ask mothers [clients] to come back for review and as well purchase the herbal shrub for body wash and oral consumption. Once she bathes and drinks that mixture/concoction, there will be no further complications” [TBA, 41 years].*

iv) Herbal uterotonic facilitates labour

The “hot” herbal uterotonic was reported to facilitate and ease labour pains and could correct breech presentation. Therefore, when mothers feel their labour progresses slowly, they can take herbal uterotonic before attending the health facility.

*“When mothers are in their ninth month, they expect labour, especially when they know about their Expected Date of Birth (EDB). However, once the EDB elapses, they don’t come to the facility; instead, they go to the TBAs for “hot” herbal uterotonic. It is taken orally and smeared on the abdomen to speed up labour, thus inducing labour early” (Healthcare Provider, 45 years).*

This healthcare provider noted that the “hot” herbal uterotonic was a proactive measure for labour induction and mainly was administered at the onset of labour. *“On the part of the herbal uterotonic, some say that it makes the birth faster and easy for them when they take it. So because of that, most of them go for it” [Healthcare provider, 33 years].*

Homebirths were common in some communities within the study districts, and the use of “hot” local herbal uterotonic was often associated with those ‘unattended’ (‘unattended’ - no skilled health care personnel in attendance) birth. However, the use of ‘hot’ herbal uterotonic could also result in roadside births:

*“We have had cases of homebirths among pregnant women. It is common among women whose labour do not keep long (precipitate labour). Also, when the “hot” herbal uterotonic is administered so early, she will give birth at home or on the roadside” [TBA, 63 years].*

xxii) Attraction of home birth for cultural reasons

While physical, economic, and cultural barriers may restrict a woman’s ability to access skilled health services, some pregnant women were reported to take pride in having a home birth and using herbal interventions due to the belief that this reflected personal strength and the ability of the woman’s body to perform as it was designed to.

*“They want to deliver in the house so that they (family/community) will know, yeah, she is a woman, and for that matter, a strong woman. That, all her deliveries took place in the house” [Healthcare provider, 48years].*

Some behaviours appeared to be intergenerational and often culturally inclined:

*“For some mothers, it is not the cost; they intentionally do it. They will tell us the abdomen did not pain for long. That the moment they felt pains, the baby was coming, so they could not get to the facility. Others say that their great-grandparents practised home deliveries, and they always have it successfully” [Healthcare provider, 59years].*

vi) Affordability of herbal uterotonics

As a component of birth preparedness in Ghana, women are expected to carry a set of items when they visit the facility to give birth. The pregnant woman is expected to purchase these prior to receiving care:

*“However, the cost I am talking about is the small items the midwives ask them to buy, such as items required during labour - rubbers, mackintosh mattress, Dettol, et cetera”. [Healthcare provider, 58 years].*

On the other hand, TBAs do not focus on the importance of these items. Therefore, women who did not intend to acquire them for the reason of cost were more likely to utilise TBAs’ care during labour.

The high cost of transportation to PHC facilities and the hospital also served as a significant barrier to women’s geographical access to facilities, thereby encouraging the use of TBAs services. It is worthy to note that TBAs reside within same communities of expectant mothers or within nearby communities. Besides, the proximity, TBAs have gained the trust of the communities and families within their jurisdictions.

“*The cost at the hospital when they seek care, and the cost of the transportation were deterrent factors. How to get transport to come to the facility is a problem. For those residing in very remote communities, mothers struggle from Kojokpere or Fian to get to Daffiama. From these communities, getting a lorry to Nadowli hospital is a problem; unless it is a market day or if her husband has a motorbike, it is always a problem. So, transportation cost is one reason for using alternative sources of care. TBAs live in the same or nearby communities of pregnant women, so they are just walk-in for their services or call on them; they need their care” [Healthcare provider, 35 years].*

### Potential adverse impacts of herbal uterotonic intake

Under the domain of potential adverse impacts, perspectives of healthcare providers and TBAs were considered and found to have many similarities. Both health care providers and TBAs recognised herbal uterotonics to be potentially potent and associated with unintended outcomes. While these themes of (i) unintended consequences and (ii) adverse outcomes was the focus for health care providers, TBAs responses also reflected the role of uterotonics in managing pregnancy and birth with two different themes identified: *risks using uterotonics to manage fertility* and *risks using uterotonics to facilitate home birth.*

### Perspectives of healthcare providers

i) Unintended consequences and adverse outcomes

Although women utilise herbal uterotonics, a Physician noted that there were no precise dosages for such herbal medications. Thus, the product is often used arbitrarily without awareness of the potential side effects on their health, sometimes with unintended consequences. These include prolonged labour and caesarean sections.

*“It gives us, one a prolonged labour. It is an issue, and sometimes mothers also report to the facility with antepartum haemorrhage. Why? Because of the premature contractions after administering the herbal uterotonic. At the time that the natural contractions are supposed to set in on their own [self-induced]. They will be in the contractions, and their contractions cease when they enter the facility. I will add that this has even contributed to more of our caesarean sections (CS), when local herbs from TBAs are given to them [expectant mothers]. Some mothers have reported bleeding cases, some preterm labour, infections, and other obstetric problems; some even lost their babies through the local herbs because it has no dosage. The TBAs just fetch for them to go and take” [Healthcare provider, 39 years].*

A midwife found that poor dilatation caused by herbal uterotonic consumption could lead to uterine rupture during labour: “*That one the cervix may not be dilated, then when the TBA gives it, she will be having the contractions, and the uterus can rupture. They are unaware that their uterus can rupture” [Healthcare staff, 36 years].*

Some clinicians noted from their routine monitoring [of maternal health service delivery] that the herbal uterotonic’s ability to induce labour might create health problems for the foetus, including stillbirth and birth asphyxia:

*“In such an instance, the expectant mother gets irregular contractions, severe ones, and when they do not get to the facility in time for immediate care, the foetus’ breath will reduce. So, the child can get asphyxia. In case it is a home birth, the baby may die” [Healthcare provider, 45 years].*

*“The herbal uterotonic may also contribute to preterm births, leading to stillbirth” [Healthcare provider, 26 years].*

*“Therefore, the midwives educate pregnant women to understand that herbal uterotonic brings about vigorous or frequent contractions with poor cervical dilation, resulting in Caesarean birth or loss of both the mother and baby. They are educated more on these harmful practices and beliefs on childbirth” [Healthcare provider, 36years].*

### Perspectives of traditional birth attendants

ii) Risks using herbal uterotonics to manage pregnancy and birth

Although the analysis shows that herbal uterotonic intake in pregnancy and birth could lead to obstetric problems, including preterm births, stillbirth, and maternal deaths, TBAs also shared views on the potency and incorrect administration of herbal medicines to cause abortions. For example, some women may seek the product illegally to terminate the pregnancy. Such decisions may contribute to poor maternal outcomes if the woman experiences complications.

*“The uterotonic for labour is usually very ‘hot’ in terms of efficacy. They are typically taken when labour starts. The powdered ‘charcoal-like’ one is mixed with a drink for prenatal care medication. Still, a little drop of salt pitter [Potassium nitrate] is added to reduce the herbal uterotonic’s side effects on the woman and the baby. Wicked women do come seeking the “hot” herbal uterotonic so that they could abort their babies illegally, but I have never given any of such women the medicines” [TBA, 70years].*

Even though pregnant women were warned during ANC against the use of herbal uterotonic and home birth, there was a reported case of maternal death of a TBA’s client who insisted on having a home birth using an herbal uterotonic:

*“They [Ghana Health Service] warned me against providing herbal uterotonic to expectant mothers. Their chief complaint was that it was reported some women who administered herbal uterotonic had poor pregnancy outcomes or outright death of the mother. The death occurred when the said mother, who had had a previous CS birth, declined to medical counsel, and administered the hot herbal uterotonic to deliver at home. So, there was a rupture of the uterus, and she failed the attempted home birth. When the family rushed her to the hospital, they found that she suffered profuse uterine bleeding. She died in the process, together with the baby” [TBA, 65years].*

Some TBAs understood that when the “hot” herbal uterotonic is administered too early during the onset of labour, the mother may not be able to reach the healthcare setting before birth. While the herbs have the potency to induce labour, their [herbal uterotonic] intake could also lead to roadside births (born-before-arrival at health facility) and their associated risks.

*“However, the ‘hot’ herbal uterotonic is administered when labour commences, to facilitate the progress of the labour… also, when the ‘hot’ herbal uterotonic is administered so early, the pregnant woman will give birth at home or on the roadside” [TBA, 49years].*

## Discussion

### Summary of findings

The study aimed to explore women’s use of herbal uterotonics from the perspectives of traditional birth attendants and allopathic healthcare providers in rural North-Western Ghana. A total of 43 women and men participated in individual face-to-interviews that lasted for approximately 16 minutes. We found that the confidence placed on traditional birth attendants care, health system challenges related to cost of healthcare utilisation, the challenges faced by the National Health Insurance Scheme (NHIS) to provide adequate essential medicines to health facilities, pregnant women’s perceived benefits of using herbal uterotonics to address obstetric problems, affordability of TBAs care, and pregnant women’s intrinsic potential hunger and pride in having culturally appropriate maternal healthcare were key reasons for the continued use of herbal uterotonics. The findings have been put into two domains and nine themes. We have thus discussed the findings based on the two domains.

#### Perceived rationale for herbal uterotonic intake

First, it was found that pregnant women and their families placed substantial trust on the services of traditional birth attendants. Trust, in this case, was explained to mean a firm belief in the reliability of the services provided [9], and how these services are provided. Trust in TBAs’ midwifery skills and the firm belief in the herbal uterotonics have attracted pregnant women. Meanwhile, although herbal uterotonics have not been proven to be efficacious in addressing maternal health problems in the study area, TBAs continued to provide care to pregnant women. It was admittedly the sole form of medication for all ill-health conditions that women present to them. Two decades ago, TBAs formed part of the continuum of maternal healthcare provision in Ghana [9]. Their inclusion in maternity care was primarily attributed to inadequately qualified healthcare providers and unaffordable health care services, particularly during an obstetric emergency. Therefore, some selected TBAs were trained and equipped with essential knowledge and skills to assist birth in the latter part of the twentieth century, resulting in TBAs gaining the trust of communities within their operational jurisdictions and beyond [27]. Nevertheless, in other low-middle-income settings such as Uganda [28], Kenya, Nigeria, and Lao, the People's Democratic Republic, community-level trust on TBAs and the continued use of their services was reported [29, 30]. Notably, the literature from Uganda has shown that pregnant women preferred TBAs’ care due to their self-acclaimed spiritual ability to foresee and avert potential dangers, which often culminates to herbal uterotonic intake [28]. This then suggests that women should be engaged more appropriately in the healthcare facilities to build trust as an approach to increasing skilled service utilisation for optimised maternal outcomes in rural Ghana. Indeed, the trust issues in the health system could be restored by using media outlets and antenatal care avenues to educate women on the risks associated with TBAs’ medicines and services and discourage TBAs from providing herbal uterotonics to pregnant women. From healthcare providers’ views, a necessary challenge addressing women continued use of herbal uterotonics is because TBAs reside in the same communities or neighbourhoods as the pregnant women, which gives them proximity compared to the health facilities. Therefore, the community surveillance function of the CHPS concept in Ghana must be given ample resource support to empower Community Health Nurses and Midwives to engage pregnant women in a more holistic and culturally appropriate manner at the home level through their prenatal journey.

Further to this, the cost of receiving professional maternal care was found as a significant barrier to and an antecedent to herbal uterotonic uptake. From healthcare providers perspectives, the cost of care which deterred mothers comprises; transportation, feeding when admitted, purchase of birth kits, medicines, etc. The NHIS prescription policy limited lower-level primary care facilities from administering certain medicines, especially antibiotics, to newly delivered mothers [19]. Indeed, the non-availability of essential medicines to manage labours and post-childbirth care, at the CHPS level compelled some referrals. However, TBAs non-conventional herbal uterotonics were relatively affordable when compared with allopathic medicines. All these partly underscore the increased intake. This finding confirms previous findings in Ghana and other sub-Saharan African countries [8, 31]. In fact, an earlier study in the study area found that some families and expectant mothers failed to honour referrals due to the cost-related factors [19].The numerous challenges associated with Ghana’s National Health Insurance Scheme, which include unauthorised charges on patients [32], and increased out-of-pocket payments (OOPs) for medicines and services, and delayed reimbursement of claims were crucial factors affecting maternal health services delivery and the motivation for the patronage of non-conventional herbal uterotonic [32]. This policy limitation has resulted in unnecessary referrals of women in labour to district hospitals, which has caused maternal mortalities and stillbirths [19]. In fact, for some women, instead of honouring referrals, women would often choose home birth or use non-conventional herbal uterotonics in attempt to treat complications or faciitate home birth [8]. This then suggests urgent calls for the health system and financing reforms to enhance service delivery, build lower level resource capacity, contain the cost of care, and provide adequate financial protection, especially for expectant mothers, to curb the utilisation of the TBAs services.

Furthermore, supplications from participants have demonstrated that pregnant women perceived that this non-conventional herbal uterotonic was multi-purposed, which could assure the user of a certain degree of healthy life through the pregnancy and can facilitate labour. It is more so because, from the findings, most women in the study area had a high desire to give birth at home to avoid the many barriers to accessing care in Ghana’s health system. Thus, expectant mothers’ resort to this herbal uterotonic to achieve these maternal health aims. This has been common in Ghana and similar sub-Saharan African countries [12, 15]. In Southern Ghana, for example, Aziato and Antwi presented recommendations suggesting that expectant mothers’ family members, relatives’ recommendations and increased advertisements on the electronic media concerning the potency of certain herbs to treat maternal health problems and induce labour have motivated non-conventional herbal uterotonics use [21]. Besides these, it was reported that the testimonies from previous and current users of specific herbal medicines in pregnancy also attracted women to patronise them. Admittedly, most of them trusted the recommended medicines of TBAs [21]. Thus, the present findings agree with the literature on non-conventional herbal uterotonic use. Besides, the finding on increased uptake of herbal uterotonic corroborates with previous findings from Southwest Nigeria [29]. In this Nigerian study, for example, Okafor and colleagues found that “aseje”, (a special concoction, mainly herbs) and “agbo” (liquid herbal concoction) were a significant attraction for pregnant women's use of TBAs’ maternal services. They believed these herbs can help to prevent any complications during pregnancy and labour and keep pregnant women healthy. They also receive ‘agbo’ to have a small baby and easy childbirth [29].

Moreover, participants believed that pregnant women also perceived herbal uterotonics as a solution to prolonged labour and helped remedy obstetric problems and preserve cultural heritage. Others perceived that herbal uterotonics have the potency to address obstetric issues. The perceived efficacy of herbal uterotonics was a motivator for women’s decision to utilise TBAs' care in addressing health complications. Meanwhile, healthcare providers noted that TBAs herbal uterotonics do not have an appropriate dosage, and undocumented side effects. The absence of correct dosage suggests that clients could abuse or misuse them. Nevertheless, the findings on pregnant women using herbal uterotonics and TBAs care more generally demonstrate that a more complex phenomenon affecting skilled maternal health service use in many communities may exist than anticipated. In this study, women’s willingness to demonstrate their prowess through homebirth has been widely reported in sub-Saharan Africa [12, 15].

Nevertheless, the present study’s findings have confirmed the extant literature in Ghana, Ethiopia and LMICs, more broadly [30, 33]. For example, in Ghana, women reportedly used herbal uterotonic to have a home birth because of fear of undergoing caesarean section (CS), where labour does not progress satisfactorily [19]. In Ethiopia, of the 250 pregnant women who participated in the study, the majority used herbal uterotonic, ginger, garlic, among other herbs, during pregnancy to correct obstetric problems [33]. In Lao, the People’s Democratic Republic (PDR), Sychareun and colleagues reported mixed findings. For instance, maternal care practices at the health facilities, including a horizontal birth position, lack of privacy, male staff’s presence, and the desire to have family hold them during childbirth, were solid motivations for preferring non-conventional herbal uterotonics [30].

In comparison, some women in that study have expressed their heartfelt desires to obey traditional norms associated with birth [30]. This finding suggests a potential gap in health education to offset wrong unverifiable claims of perceived efficacy of herbs and create public awareness to empower pregnant women in healthcare decision-making. Overall, the findings portray existential health system problems about financing, staffing capacity, and influence of childbirth traditions/culture.

#### Perceived consequences of herbal uterotonic use

While participants itemised potentially good reasons for the increased use of herbal uterotonics and TBAs services, they also recognised that there might be unintended consequences with birth occurring in transit between home and healthcare facilities, increasing the risk for the mother and the baby.

First, both TBAs and healthcare provider participants perceived those women who patronised herbal uterotonics during pregnancy were more likely to use them during labour. At the same time, they were also cognisant of the risks, and noted that herbal uterotonics posed significant risks for women and their babies by increasing the risk of preterm birth and birth asphyxia and other adverse health outcomes. This corroborates previous studies. In one of those studies, women who used herbal uterotonics during pregnancy repeated the same during labour. However, stillbirths were recorded among users of these herbal substances [19].

Also, the interviews with TBAs and healthcare providers reported that expectant mothers who administered herbal uterotonic were prone to complications such as postpartum haemorrhage (PPH), rupture of the uterus, birth asphyxia, puerperal pyrexia, and maternal mortality. These results correspond to the findings of earlier studies by Sumankuuro and colleagues assessing the causes of maternal deaths in Ghana, which have demonstrated that maternal deaths, prolonged labours and cases of fistulas were recorded among users of herbal uterotonic [19]. Although nearly all medicines have side effects when wrongly used, there are often counter medications to reduce the adverse impact. However, the findings have shown that TBAs do not have alternative solutions and measures to counteract any ill-health condition associated with herbal uterotonic consumption. Its continued use may increase the risk for maternal health interventions primarily due to severe shortages of obstetricians and skilled midwives and the undeveloped emergency referral system in northern Ghana. This, in turn, may have negative repercussions for achieving the overall maternal goals and targets.

#### Implications for policy and practice

Overall, TBAs viewed the use of this herbal uterotonics (‘mansugo’) favourably, observing multiple benefits for the mother during pregnancy and labour. On the other hand, healthcare provider participants were more likely to believe that herbal uterotonic intake in pregnancy and birth could be harmful to women and infant health, including being associated with postpartum haemorrhage, uterine rupture, preterm birth, and could lead to lifetime disabilities and death of both mother and baby.

Thus, the findings depict a complex cultural and behavioural phenomenon linked to the breakdown of health systems, geographical complexities related to community culture, societal influence, and the TBAs desire to maintain their relevance in the continuum of maternal care. Specifically, preferences for care from TBAs and distrust of the healthcare system coupled with laxity in quality maternal service delivery and utilisation accounted for women and families’ decisions. Maternal literature reveals that herbal uterotonics may have some hormonal benefits to pregnant women, solve pregnancy problems, and some potency for labour induction. However, the actual side effects of the herbs and what doses were adequate to address a given situation is not known. There is a clear knowledge gap in the constituents of the products as espoused by the participants in this study. Until answers are provided for these concerns related to this herbal uterotonic consumption, physicians and licensed herbal medicine practitioners must investigate the safety and effects of herbal uterotonic intake on the women’s health. The findings have shown that TBAs make maternity care more affordable, capturing the difference between healthcare on one side and cost and access to essential medicines on the other strand. Thus, if women must use a TBA due to the cost of care, they must use herbal uterotonics. So, choices are limited. This is important because understanding factors at play can influence strategies to lead to behaviour change.

Key educational messages for women and the broader community should focus on moderating the use of herbal uterotonics and highlighting the potential negative effects of their overuse in pregnancy and labour and for the newborn.

#### Strengths and limitations of the study

The diversity of views is one of the study’s strengths. However, given the research’s breadth, the results cannot be considered to provide a comprehensive understanding of the use of herbal uterotonics as the design and sample size used did not allow for accurate measurement of the prevalence of uterotonic use in the population.

Nonetheless, the congruence of results suggests that the diverse views obtained from participants are sufficient to guide further research into the use of these interventions and to inform policy formulation and maternal health service delivery in similar communities, particularly in the hard-to-reach populations in northern Ghana. More detailed research into the use of herbal uterotonic is recommended, given its ongoing use and the considerable barriers to using formal maternal health services that continue to exist in rural locations. This will underpin strategies to minimise harm while achieving long-term cultural change related to the use of TBAs’ maternal healthcare services and herbal uterotonics.

## Conclusion

The findings have suggested that the intake of non-conventional herb uterotonic is widespread in the study area. However, the reasons for the intake are complex and include  healthcare cost, desire for homebirth, unawareness of the negative effects of such substances, perceived way of addressing obstetric problems and cultural undertones, among others. However, the diverse views of participants also point to the fact that the intake of these herbal uterotonics can cause poor health outcomes. This thus calls for stakeholder engagement, and policy dialogue to address the pervasive administration of non-conventional herbal uterotonics. We also encourage research into the constituents of ‘mansugo’ and the potential benefits and adverse effects. We recommend qualitative studies involving previous users of this herbal uterotonic to inform policy and healthcare provision.

## Data Availability

The datasets generated and/or analysed during the current study are not publicly available because we are still writing-up from it but are available from the corresponding author on reasonable request.

## Abbreviations

ANC: Antenatal Care
CHPS: Community-based Health Planning and Services
CHSVs: Community-based Health Surveillance Volunteers
GHS: Ghana Health Service
JICA: Japanese International Cooperation Agency
LMICs: Low-and middle-income countries
NHIA: National Health Insurance Authority
NHIS: National Health Insurance Scheme
PPH: Postpartum Haemorrhage
TBAs: Traditional Birth Attendants
UWR: Upper West Region
WHO: World Health Organisation
